# Supplementary material for: Establishment and Characterization of a Brca1−/−, p53−/− Mouse Mammary Tumor Cell Line
Source: Int J Mol Sci. 2020 Feb 11;21(4):1185. doi: 10.3390/ijms21041185 (PMC7072850; doi:10.3390/ijms21041185)
Supplement: Supplementary file 1 [file ijms-21-01185-s001.pdf]

Supplementary information

Supplementary Figure S1

A

MSC from bone marrow of FVB mouse				
Sca1	CD44	CD90.1	FLK1	F4/80
+	+	-	-	-

B

Osteogenic differentiation

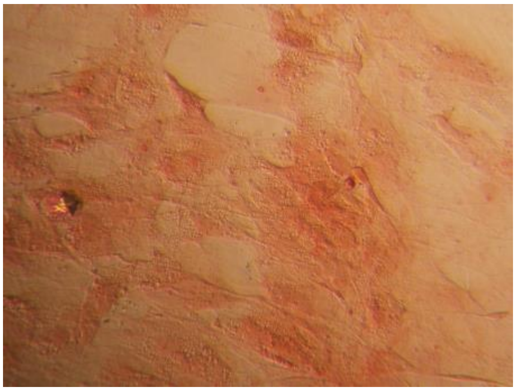

Adipogenic differentiation

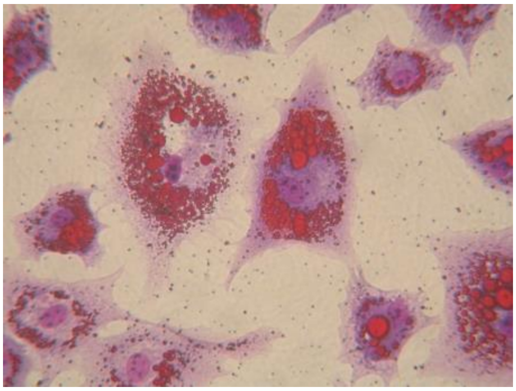

Supplementary Figure S1. Characterization of mesenchymal stem cells (MSC) derived from bone marrow of FVB mice. A) Isolated MSCs are positive to mesenchymal stem cell markers (Sca-1, CD44) and negative to CD90.1, macrophage marker F4/80 and Flk1, endothelial cell marker. B) Osteogenic and adipogenic differenciation of isolated MSCs. Extracellular matrix mineralization was evaluated by using Alizarin Red staining (osteogenic differentiation) and in case of adipogenic differentiation , cells were stained with Oil Red O to visualize the lipid droplets, and dimethyl methylene blue to stain the cytoplasm. Digital photos were taken by a Nikon Coolpix 4500 digital camera (Nikon GmbH, Düsseldorf, Germany) connected to an Olympus CK2 inverted microscope (Olympus, Tokio, Japan) with 10x objective (osteogenic differentiation) and 20x objective (adipogenic differentiation).

Supplementary Figure S2

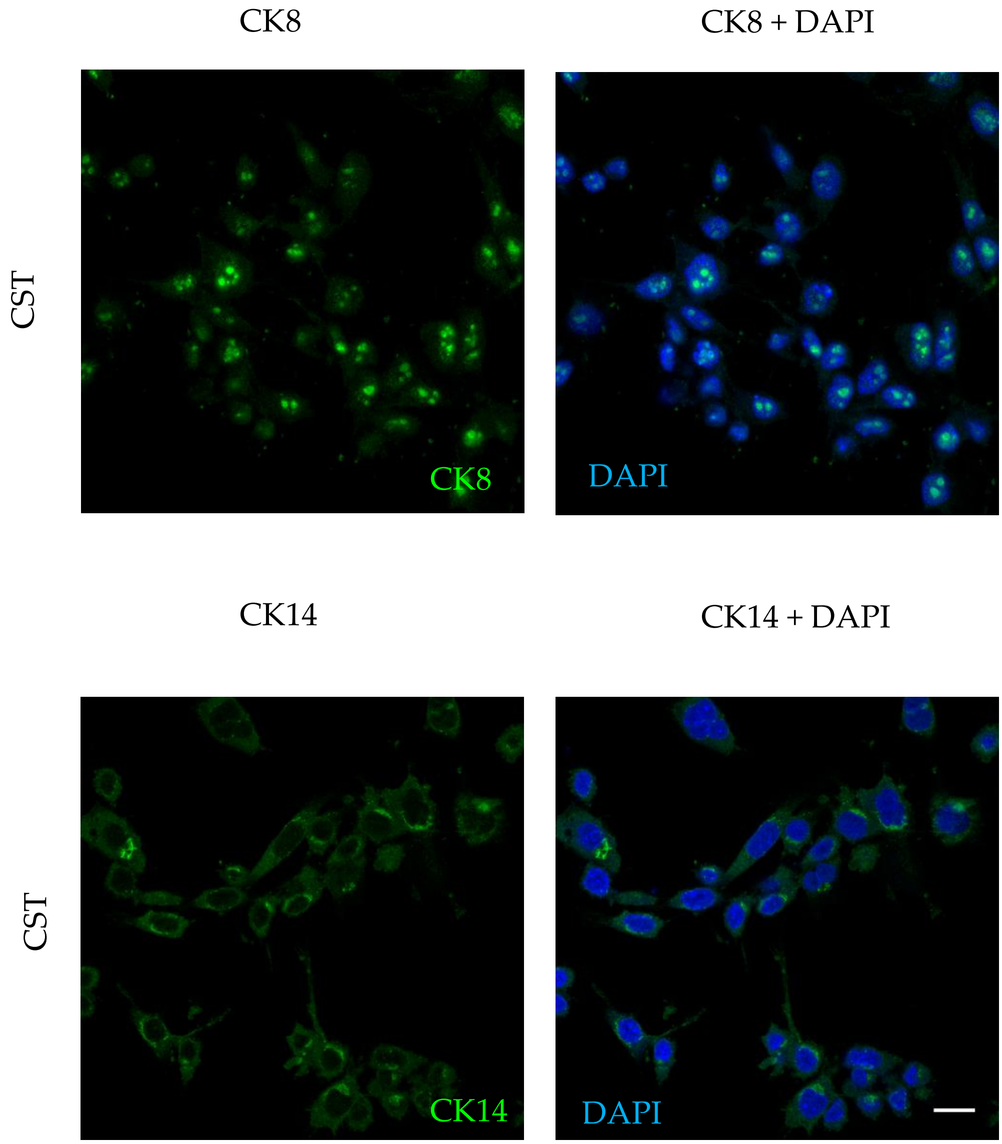

Supplementary Figure S2. Immunofluorescent detection of cytokeratins. A) Cytokeratin 8 (CK8) (Alexafluor488 – green) was detected in the cell nuclei. B) Cytokeratin 14 (CK14) (Alexafluor488 – green) localization. Nuclei were stained with DAPI. Scale bar = 20µm. Microscopy pictures were aquired using ZEISS LSM-710 system (Carl Zeiss microscopy Gmbh, Jena, Germany) with 40x/1.4 Plan-Apochromat oil immersion objective (images were processed with ZEN (Carl Zeiss microscopy Gmbh, Jena, Germany)).

Consistently with the basal-like phenotype of BRCA1-mutated breast cancers, CST cells express markers that are typical for basal/myoepithelial cells, such as the basal cytokeratin CK14 (Lakhani, Reis-Filho et al. 2005). Interestingly, we observe nuclear localization of cytokeratin 8 in CST cells. Nuclear localization of keratins has been described (Hobbs, Jacob et al. 2016) and in particular CK8 has been shown to play a role as a nuclear matrix-attachment region-binding protein involved in the organization of nuclear DNA (Spencer, Coutts et al. 1998).

Supplementary Figure S3

A

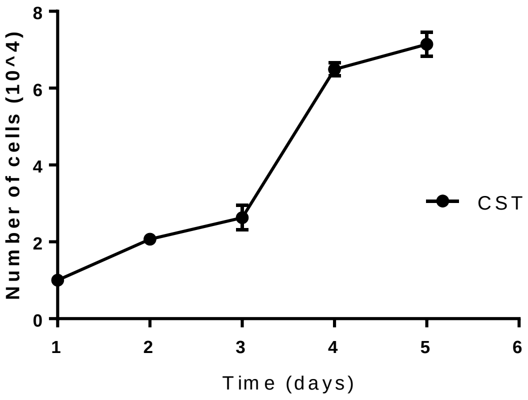

B

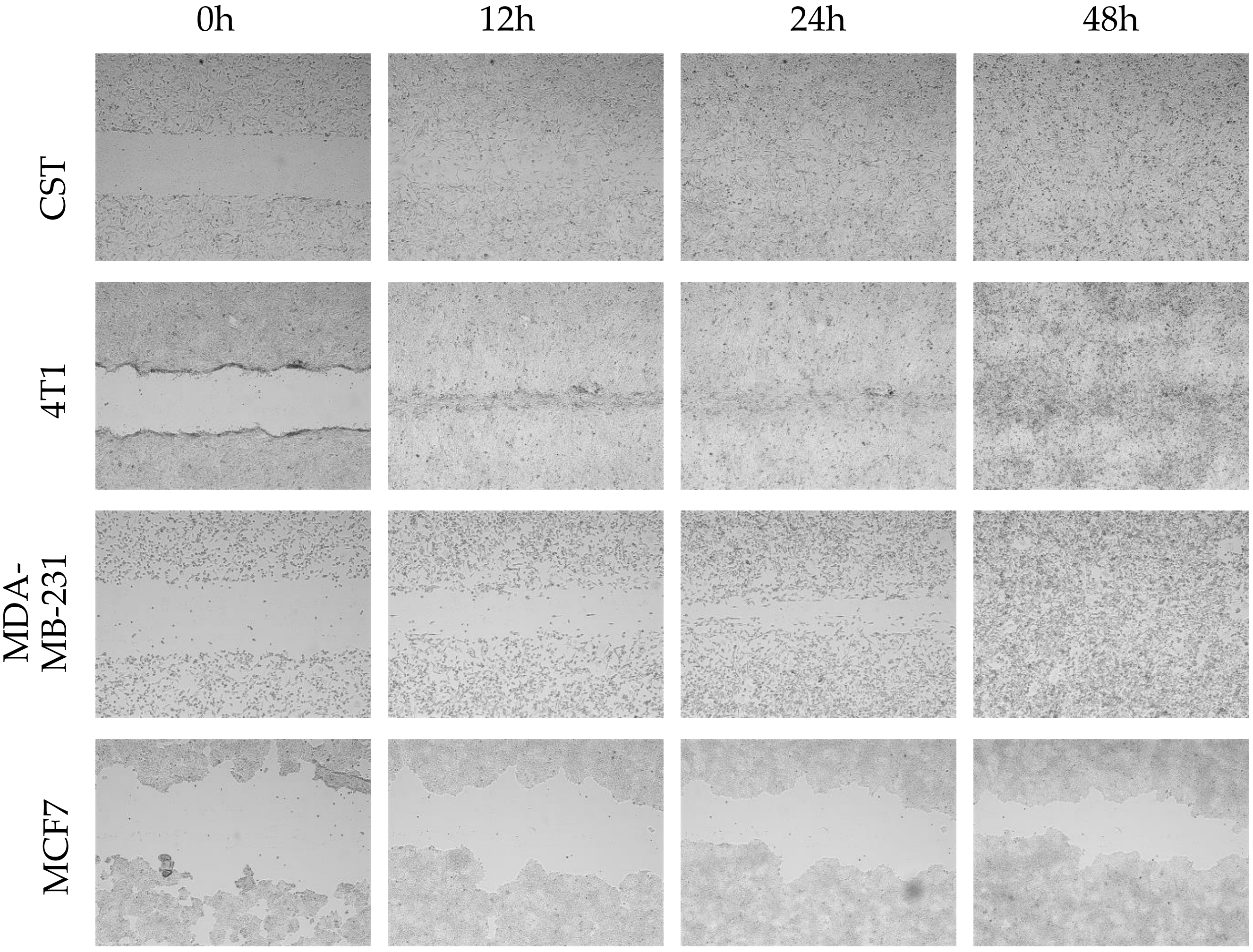

Supplementary Figure S3. A) Cell growth assay. To evaluate the growth of the established CST cell line, cells were plated at a density of 2×10^4 cells per well (24-well culture plate). Cells were counted using an automated cell counter every day for 4 days. Data represent the mean of three independent experiments (mean ± SEM). B) Wound healing. Images were acquired using JuLi™ Stage (NanoEnTek Inc., Korea) with 4x/0.16 U Plan S-Apo objective.

Supplementary Figure S4

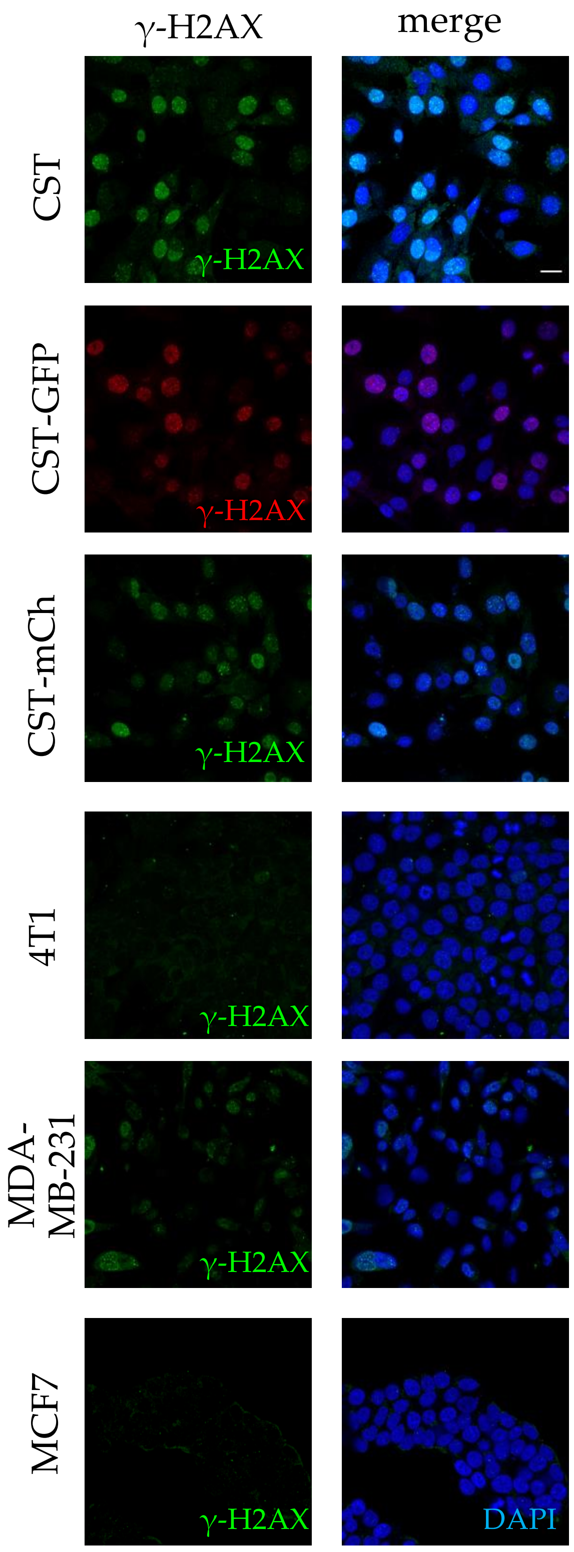

Suplememntary Figure S4. Immunofluorescence of the S139 phosphorylated form of histone H2A (H2AX) (green fluorescence (Alexa Fluor 488): CST,CST-mCh, MDA-MB-231, MCF7, 4T1, red fluorescence (Alexa Fluor 546): CST-GFP) as a measure of DNA damage. Representative confocal microscopy images show nuclear foci of phosphorylated H2AX. Nuclei were stained with DAPI (blue). Scale bar = 20μm. Microscopy pictures were acquired using ZEISS LSM-710 system (Carl Zeiss microscopy Gmbh, Jena, Germany) with 40x/1.4 Plan-Apochromat oil immersion objective (images were processed with ZEN (Carl Zeiss microscopy Gmbh, Jena, Germany)).

Supplementary Figure S5

Supplementary Figure S5. Copy number status of CST compared to K14cre; Brca1$^{F/F}$; p53$^{F/F}$ mouse tumors. In each row, ploidy levels were determined for a single chromosome. Left panels: averaged positive and negative log2 ploidy changes for the 18 mouse tumor samples from (Hollern D. P. et al., 2019), right panels: estimated coverages in 16 kb windows and the associated ploidy level predictions.

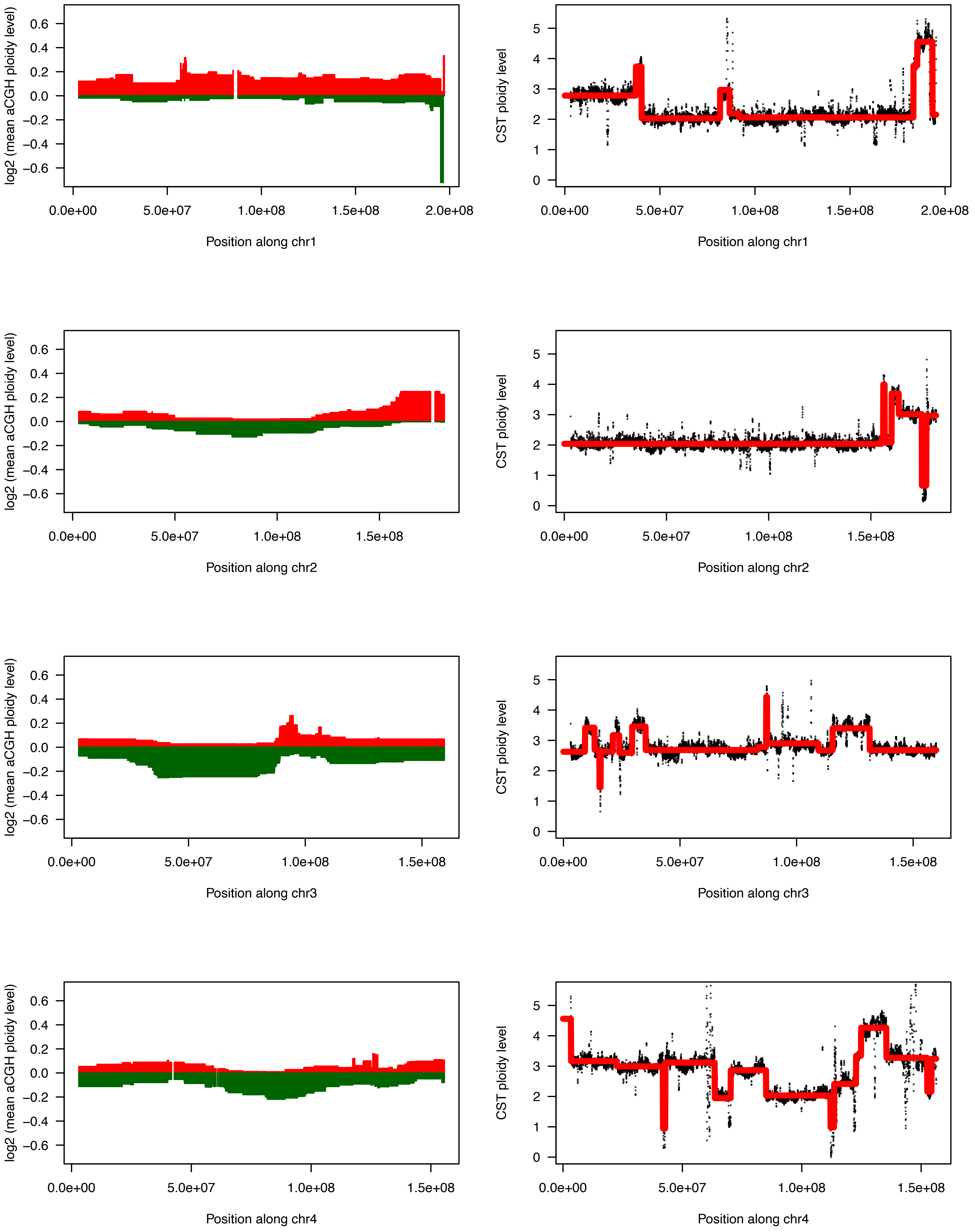
log2 (mean aCGH ploidy level)
0.6
0.4
0.2
0.0
−0.2
−0.4
−0.6
0.0e+00
5.0e+07
1.0e+08
1.5e+08
2.0e+08
Position along chr1
CST ploidy level
5
4
3
2
1
0
0.0e+00
5.0e+07
1.0e+08
1.5e+08
2.0e+08
Position along chr1
log2 (mean aCGH ploidy level)
0.0e+00
5.0e+07
1.0e+08
1.5e+08
Position along chr2
CST ploidy level
0.0e+00
5.0e+07
1.0e+08
1.5e+08
Position along chr2
log2 (mean aCGH ploidy level)
0.0e+00
5.0e+07
1.0e+08
1.5e+08
Position along chr3
CST ploidy level
0.0e+00
5.0e+07
1.0e+08
1.5e+08
Position along chr3
log2 (mean aCGH ploidy level)
0.0e+00
5.0e+07
1.0e+08
1.5e+08
Position along chr4
CST ploidy level
0.0e+00
5.0e+07
1.0e+08
1.5e+08
Position along chr4

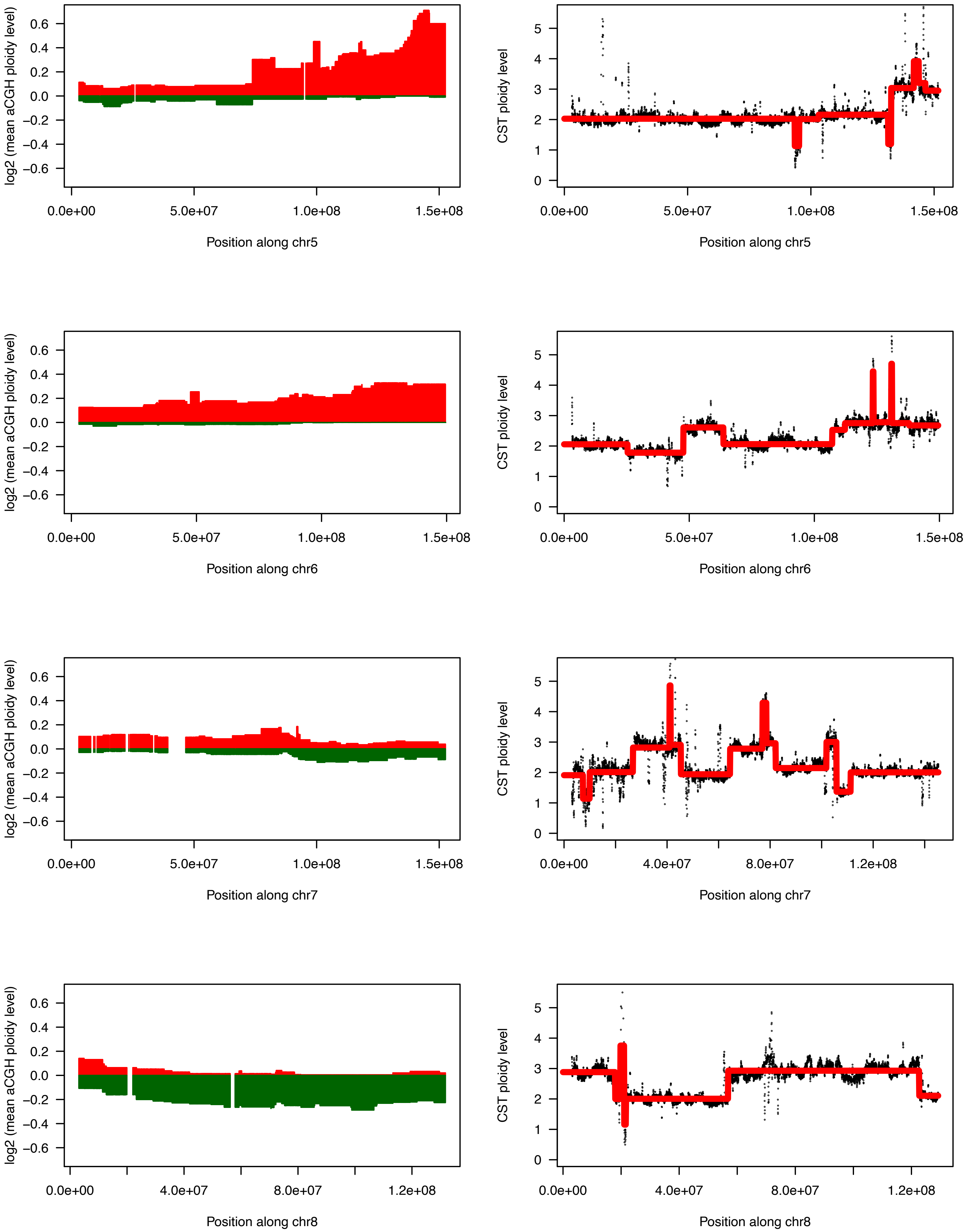
log2 (mean aCGH ploidy level)
0.6
0.4
0.2
0.0
−0.2
−0.4
−0.6
0.0e+00
5.0e+07
1.0e+08
1.5e+08
Position along chr5
CST ploidy level
0
1
2
3
4
5
0.0e+00
5.0e+07
1.0e+08
1.5e+08
Position along chr5
log2 (mean aCGH ploidy level)
0.6
0.4
0.2
0.0
−0.2
−0.4
−0.6
0.0e+00
5.0e+07
1.0e+08
1.5e+08
Position along chr6
CST ploidy level
0
1
2
3
4
5
0.0e+00
5.0e+07
1.0e+08
1.5e+08
Position along chr6
log2 (mean aCGH ploidy level)
0.6
0.4
0.2
0.0
−0.2
−0.4
−0.6
0.0e+00
5.0e+07
1.0e+08
1.5e+08
Position along chr7
CST ploidy level
0
1
2
3
4
5
0.0e+00
4.0e+07
8.0e+07
1.2e+08
Position along chr7
log2 (mean aCGH ploidy level)
0.6
0.4
0.2
0.0
−0.2
−0.4
−0.6
0.0e+00
4.0e+07
8.0e+07
1.2e+08
Position along chr8
CST ploidy level
0
1
2
3
4
5
0.0e+00
4.0e+07
8.0e+07
1.2e+08
Position along chr8

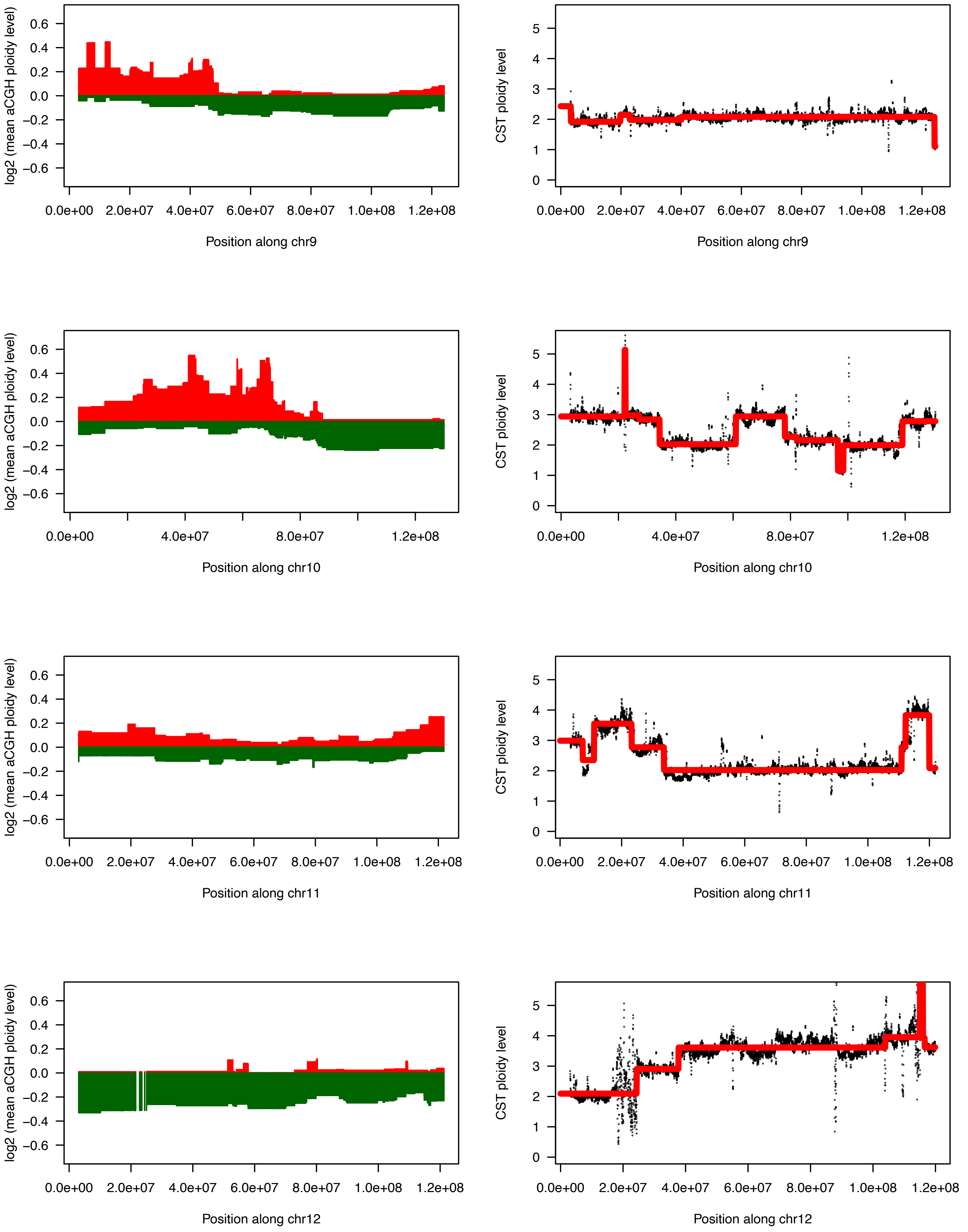
log2 (mean aCGH ploidy level)
CST ploidy level
Position along chr9
Position along chr10
Position along chr11
Position along chr12
0.0e+00
2.0e+07
4.0e+07
6.0e+07
8.0e+07
1.0e+08
1.2e+08
0.6
0.4
0.2
0.0
−0.2
−0.4
−0.6
0
1
2
3
4
5

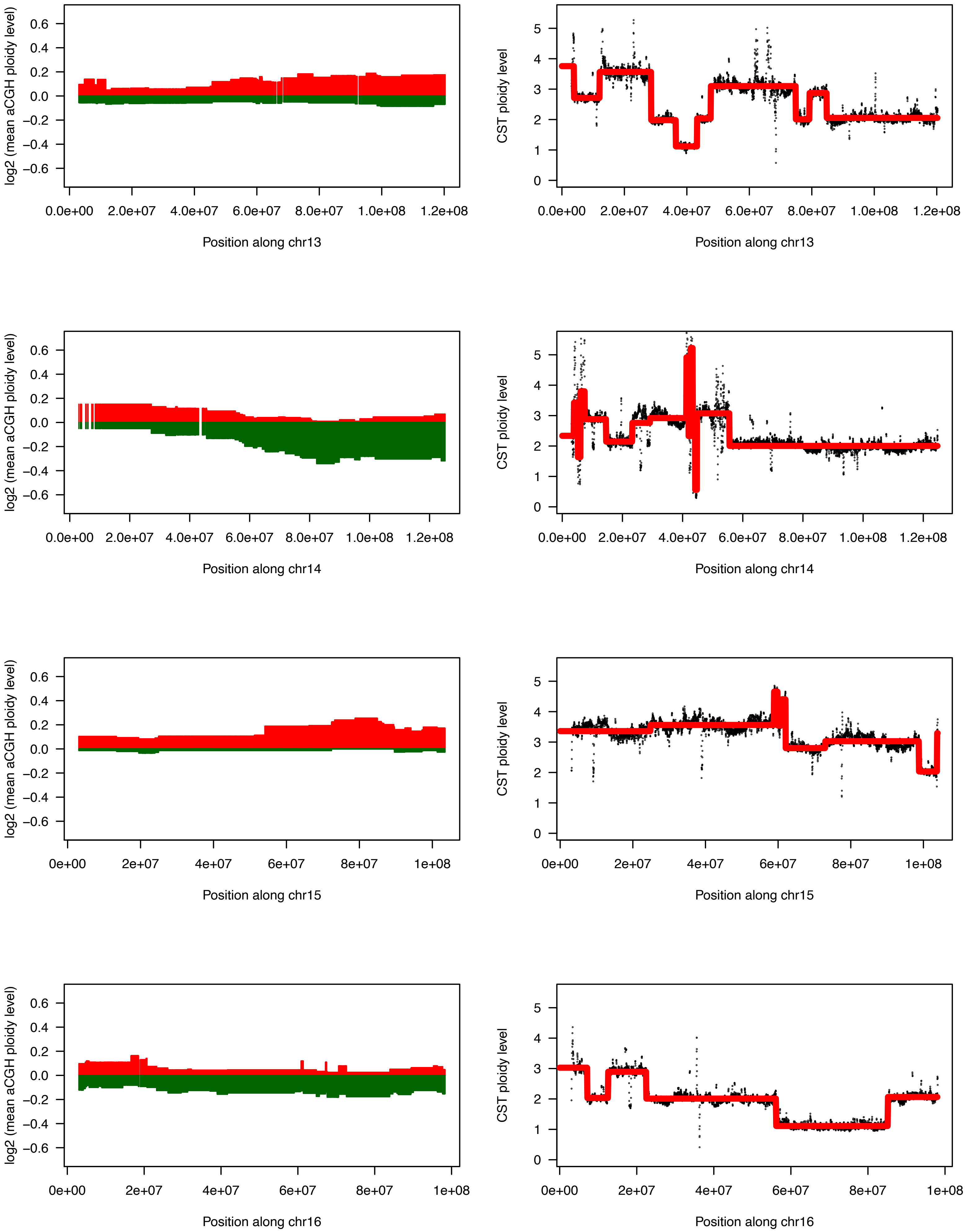
log2 (mean aCGH ploidy level)
0.6
0.4
0.2
0.0
−0.2
−0.4
−0.6
0.0e+00
2.0e+07
4.0e+07
6.0e+07
8.0e+07
1.0e+08
1.2e+08
Position along chr13
CST ploidy level
0
1
2
3
4
5
0.0e+00
2.0e+07
4.0e+07
6.0e+07
8.0e+07
1.0e+08
1.2e+08
Position along chr13
log2 (mean aCGH ploidy level)
0.6
0.4
0.2
0.0
−0.2
−0.4
−0.6
0.0e+00
2.0e+07
4.0e+07
6.0e+07
8.0e+07
1.0e+08
1.2e+08
Position along chr14
CST ploidy level
0
1
2
3
4
5
0.0e+00
2.0e+07
4.0e+07
6.0e+07
8.0e+07
1.0e+08
1.2e+08
Position along chr14
log2 (mean aCGH ploidy level)
0.6
0.4
0.2
0.0
−0.2
−0.4
−0.6
0e+00
2e+07
4e+07
6e+07
8e+07
1e+08
Position along chr15
CST ploidy level
0
1
2
3
4
5
0e+00
2e+07
4e+07
6e+07
8e+07
1e+08
Position along chr15
log2 (mean aCGH ploidy level)
0.6
0.4
0.2
0.0
−0.2
−0.4
−0.6
0e+00
2e+07
4e+07
6e+07
8e+07
1e+08
Position along chr16
CST ploidy level
0
1
2
3
4
5
0e+00
2e+07
4e+07
6e+07
8e+07
1e+08
Position along chr16

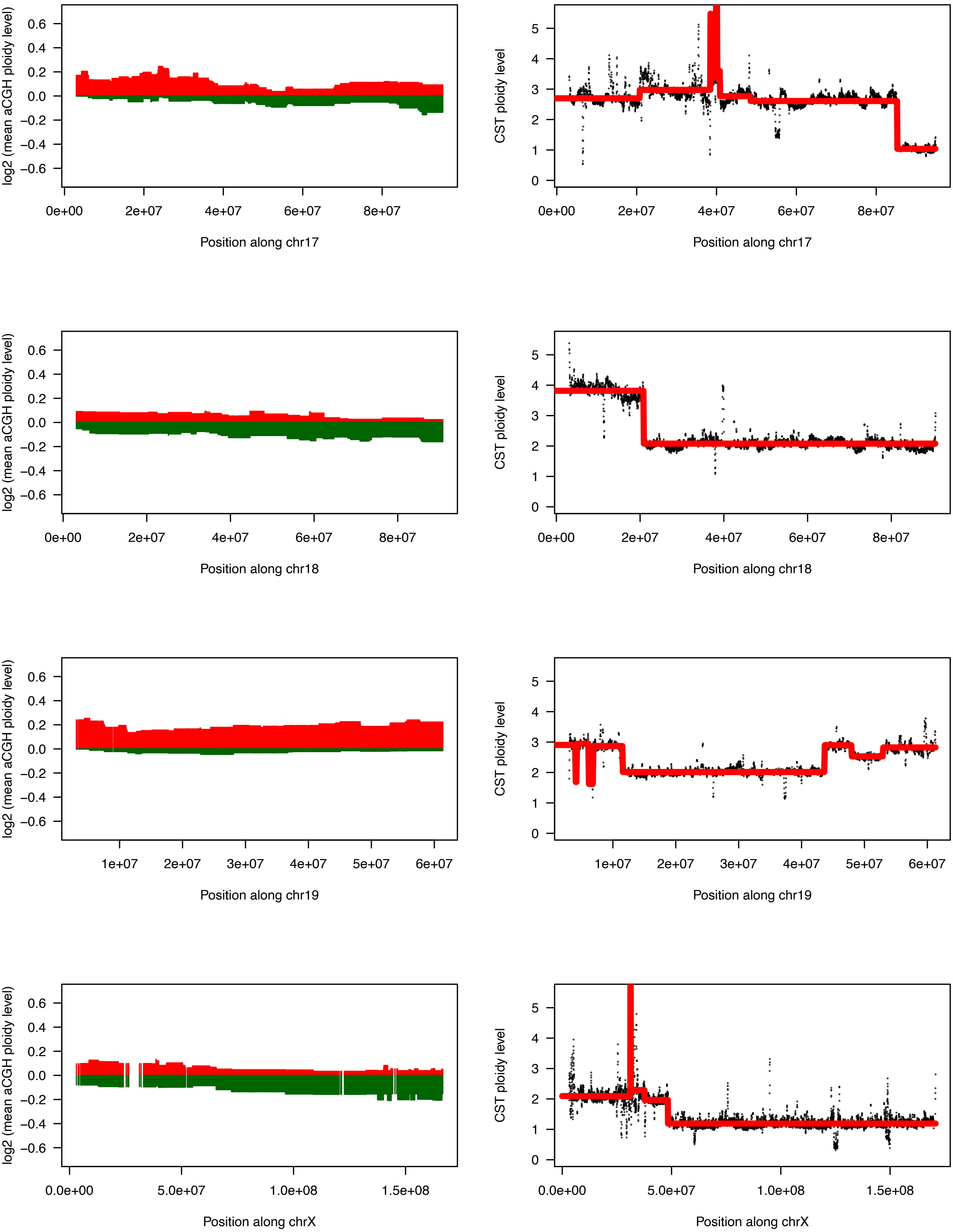
log2 (mean aCGH ploidy level)
0.6
0.4
0.2
0.0
−0.2
−0.4
−0.6
0e+00
2e+07
4e+07
6e+07
8e+07
Position along chr17
CST ploidy level
5
4
3
2
1
0
Position along chr17
Position along chr18
Position along chr18
1e+07
2e+07
3e+07
4e+07
5e+07
6e+07
Position along chr19
Position along chr19
0.0e+00
5.0e+07
1.0e+08
1.5e+08
Position along chrX
Position along chrX

Supplementary Figure S6

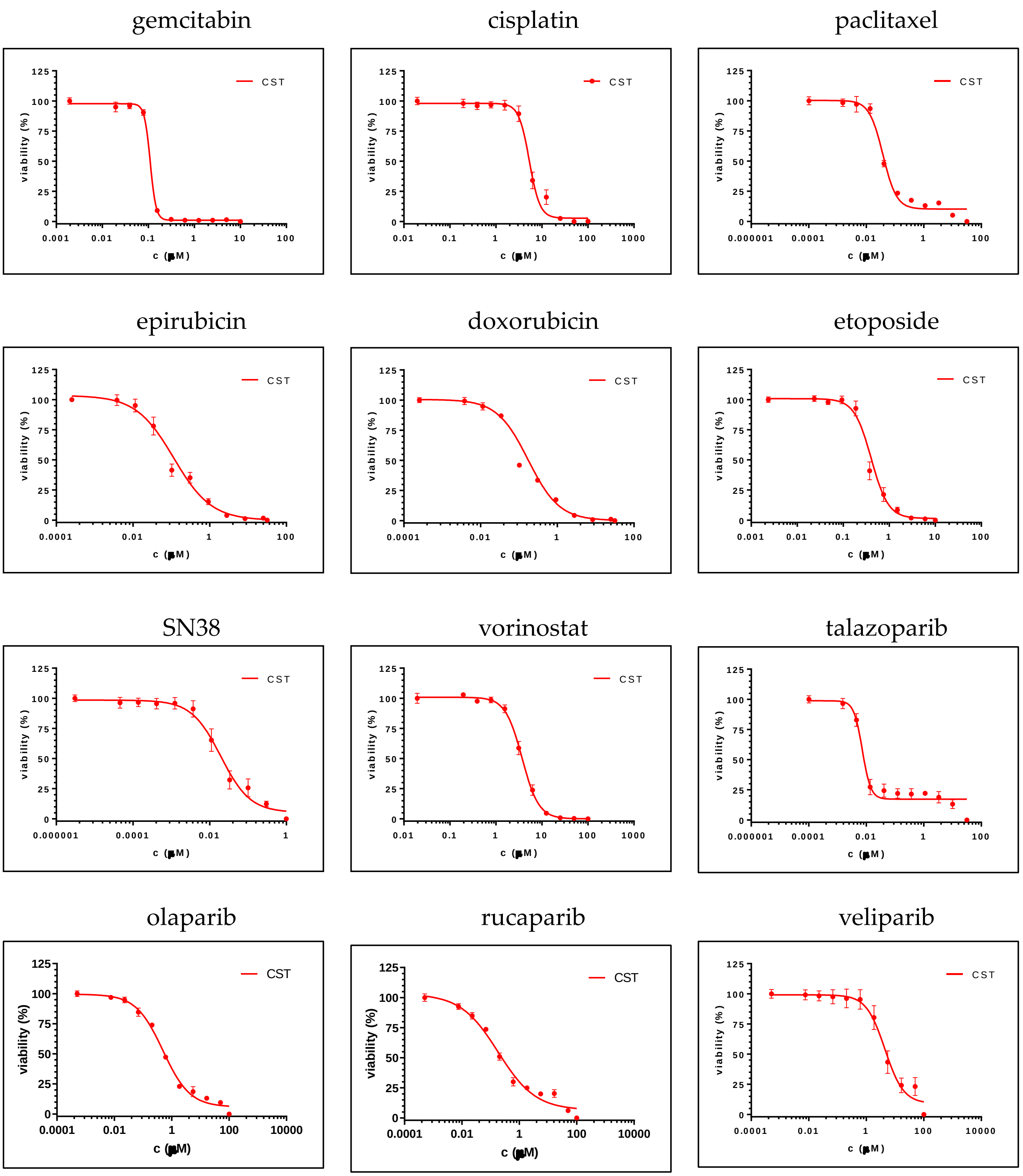

Supplementary Figure S6. *In vitro* toxicity (IC_{50}) of selected compounds, measured with PrestoBlue assay. IC_{50} values were obtained by sigmoidal curve fitting using the GraphPad Prism software.

Supplementary Figure S7

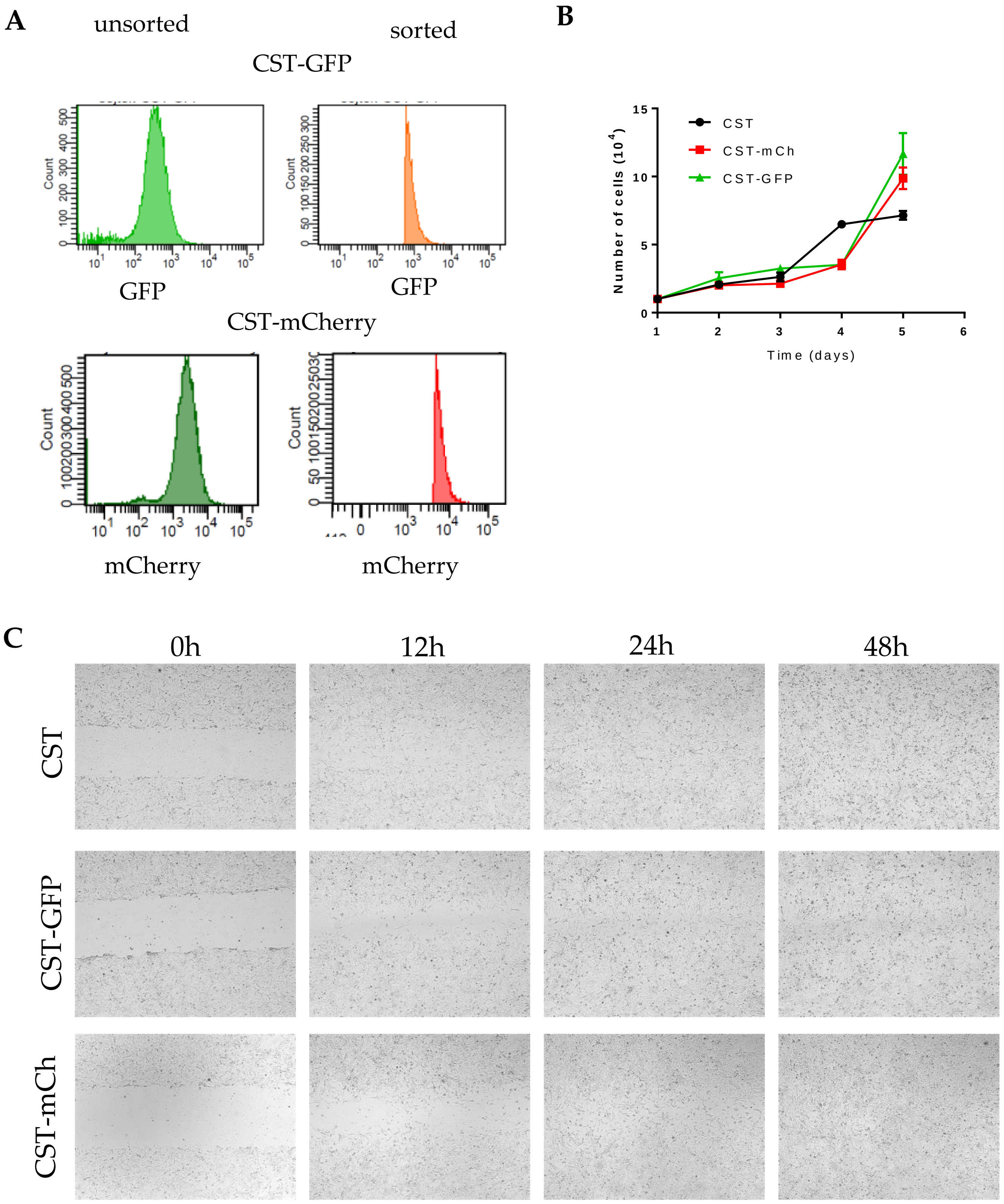

Supplementary Figure S7. A) Transduction efficiencies were measured by flow cytometry for both GFP and mCherry protein expression. After sorting transduced CST cells twice, 90% GFP+ and 95% mCherry+ cell were detected. B) Cell growth assay. To evaluate the growth of the established CST cell line, cells were plated at a density of 2 × 10^4 cells per well (24-well culture plate). Cells were counted using an automated cell counter every day for 4 days. Data represent the mean of three independent experiments (mean ± SEM). C) Wound healing. Images were acquired using JuLi™ Stage (NanoEnTek Inc., Korea) with 4x/0.16 U Plan S-Apo objective.

Supplementary Figure S8

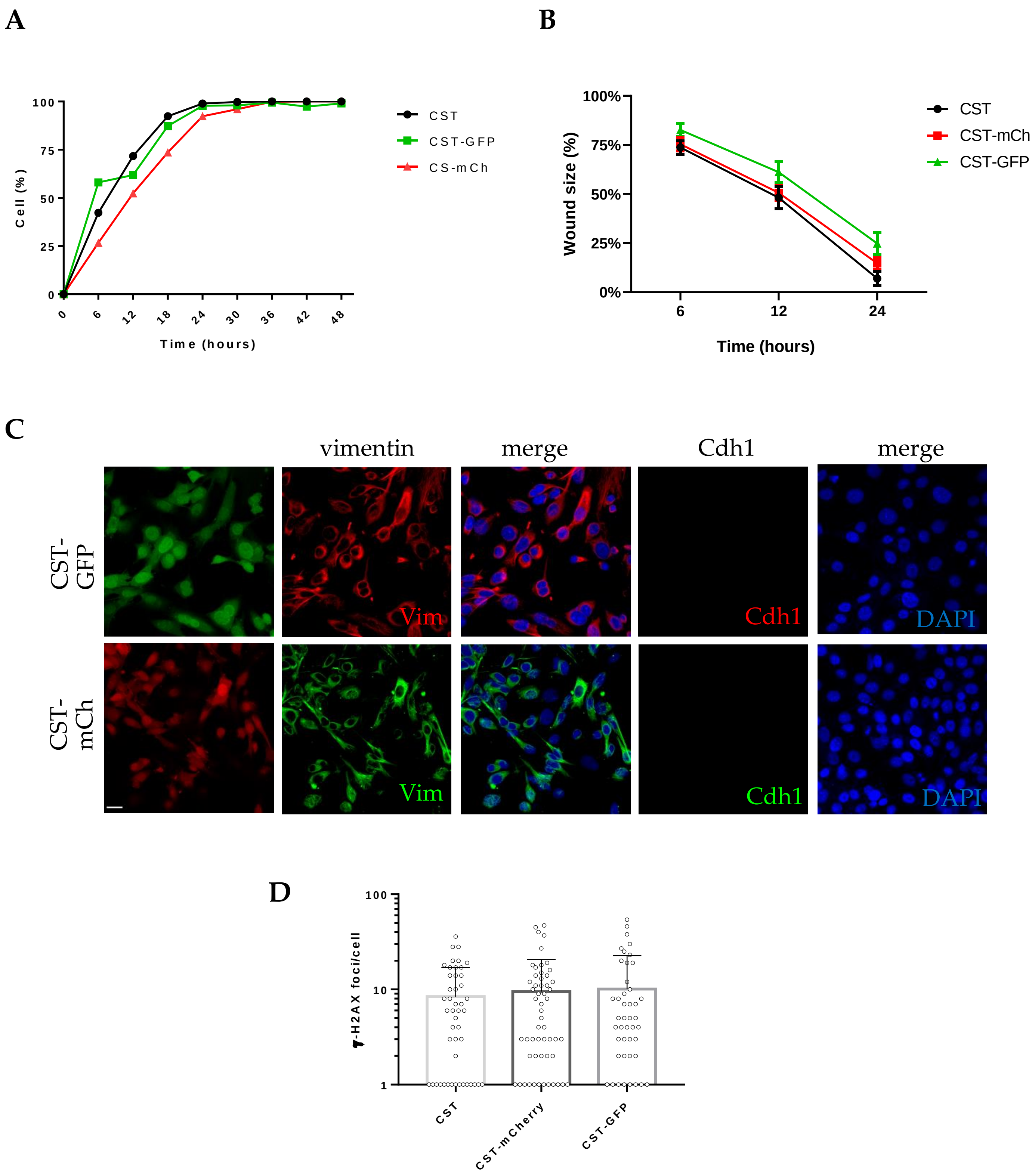

Supplementary Figure S8. Lentiviral vector-mediated gene transduction has no effect on CST cell viability, growth, motility and protein expression. A) Cell growth assay. B) Wound healing assay of parental CST cells compared to CST-GFP, CST-mCh. C) Immunofluorescent detection of fluorescent proteins (GFP or mCh) and E-cadherin (Cdh-1) (Alexafluor555-red) and vimentin (Alexafluor488 – green), nuclei were stained with DAPI. Scale bar = 20μm. Microscopy pictures were acquired using ZEISS LSM-710 system (Carl /Zeiss microscopy Gmbh, Jena, Germany) with 40x/1.4 Plan-Apochromat oil immersion objective (images were processed with ZEN (Carl Zeiss microscopy Gmbh, Jena, Germany)). D) Quantification of γ-H2AX expression. Number of γ-H2AX loci per cell nucleus are presented as mean values ± SEM.
